# Analysis of drug resistance in pulmonary tuberculosis patients with positive sputum tuberculosis culture in Northeast China

**DOI:** 10.3389/fphar.2023.1263726

**Published:** 2023-09-25

**Authors:** Li Sichen, Wang Rui, Yang Yue, Liu Xin, Cui Youbin, Tang Ze, Cai Hongfei

**Affiliations:** ^1^ Department of Thoracic Surgery, The First Hospital of Jilin University, Changchun, China; ^2^ School of Public Health, Jilin University, Changchun, China

**Keywords:** pulmonary tuberculosis, drug resistant, multidrug-resistant, retreated, initially treated

## Abstract

**Objective:** The objective of this study is to determine the drug resistance status of pulmonary tuberculosis patients in Jilin Province.

**Methods:** A retrospective survey was conducted on 395 sputum culture TB-positive patients admitted to the tuberculosis hospital in Jilin Province in 2019. Sputum samples were cultured in acidic Roche medium. Drug sensitivity testing was conducted using the proportional method. Sensitivity was reported if the percentage of drug resistance was less than 1%, and resistance was reported if the percentage was ≥1%. Statistical analysis was performed using SPSS 22.0.

**Results:** 395 tuberculosis patients with positive sputum tuberculosis culture were included in the study, with 102 being initially treated and 293 being retreated. The study population consisted of 283 males and 112 females. Sex, age, nationality, occupation, marital status, diabetes comorbidity, initial treatment, normal health status, BCG vaccine vaccination, smoking, and alcohol consumption were considered as factors that may affect the rate of multidrug resistance. And only the history of treatment (initial treatment) was associated with multidrug resistance (*p* = 0.032). This indicates that retreatment is the most significant risk factor for the occurrence of multidrug resistance in tuberculosis. The multidrug resistance rate in retreated patients is 3.764 times higher than that in initially treated patients.

**Conclusion:** The prevalence of multidrug-resistant is higher in retreated patients compared to initially treated patients in the study population. Multidrug resistance is only associated with the treatment history (initial retreatment) and not with other factors.

## Introduction

Tuberculosis is an infectious bacterial disease caused by *Mycobacterium tuberculosis* (Mtb). The respiratory system is usually the first to be affected, but it can also cause damage to other tissues (Holmes et al., 2017). The gastrointestinal system, lymphatic network, skin, central nervous system, musculoskeletal system, and reproductive system are among the most frequently involved organ systems (Adigun and Singh, 2023). According to a report from the World Health Organization (WHO) in 2017, the global estimated incidence rate of tuberculosis had been decreasing by 1.5% annually since 2000 (Jilani et al., 2023). It was predicted that in 2021, there will be 780,000 new tuberculosis patients in China (compared to 842,000 in 2020 and 833,000 in 2019), and the incidence rate of tuberculosis had been declining in China since 2000 (Bagcchi, 2023) Among the 30 countries with a high burden of tuberculosis, China’s estimated incidence rate (780,000) was lower than that of India (2.95 million) and Indonesia (969,000), ranking second in 2020 and third in 2021 (Bagcchi, 2023). However, despite global efforts to eradicate tuberculosis, the incidence rate and mortality of the disease remained high worldwide. Factors such as the increasing elderly population, the AIDS epidemic, and the influx of people in certain regions have contributed to the high infectivity, drug resistance, and regional differences associated with tuberculosis (Chakaya et al., 2021; Jali et al., 2022). Drug-resistant tuberculosis was not only a significant public health issue but also a serious social problem (Alame Emane et al., 2021; da Silva et al., 2022). Since the onset of the COVID-19 pandemic in 2019, the human immune system has been negatively impacted, resulting in a higher occurrence and reoccurrence of tuberculosis (Tadolini et al., 2020).

Research has shown that there is widespread resistance to anti-tuberculosis drugs. The prevalence rate of resistance to Isoniazid (INH) and Rifampicin (RIF) is significantly higher than previously reported, and there is still a high proportion of newly diagnosed cases with multidrug-resistant tuberculosis (Reta et al., 2022). Drug-resistant tuberculosis has increased the burden of global antimicrobial drug resistance, resulting in significant medical care expenditure and resource consumption in affected countries (Liang et al., 2022; Liebenberg et al., 2022). The world is currently facing severe challenges such as the dual infection of TB bacteria and the coronavirus, as well as HIV. Therefore, the prevention and control of tuberculosis remains a crucial task (Shariq et al., 2022; Yang et al., 2022).

Being one of the three northeastern provinces in China, Jilin Province was also grappling with respiratory diseases. This study aims to analyze the drug resistance of sputum culture-positive patients in the tuberculosis hospitals of Jilin Province. It will observe and analyze the drug resistance of pulmonary tuberculosis in northern China and investigate the factors related to drug-resistant bacteria. The findings will serve as a foundation for rational drug use in clinical settings and the development of tuberculosis prevention and control strategies.

## Methods

### Materials and methods

#### Data sources and experimental methods

##### Data sources

A total of 395 sputum culture-positive TB patients admitted to the tuberculosis Hospital in Jilin Province were selected in 2019. Among them, 102 cases were initially treated and 293 cases were retreated, with 283 males and 112 females. Test Methods According to the requirements of the Bacteriology test procedure for tuberculosis diagnosis issued by the China Anti-TB Association, sputum samples were inoculated into acidic Roche medium for culture. Drug sensitivity testing was performed using the proportional method (Habimana-Mucyo et al., 2023). If the drug resistance percentage is less than 1%, it is reported as sensitive (S); if the percentage is ≥1%, it is reported as resistant (R). The drug resistance percentage is calculated as follows: (number of colonies growing on drug-containing medium/number of colonies growing on control medium) × 100% (Getnet et al., 2017).

### Related definitions

Determination of Initial and Secondary Treatment, as well as Resistance to Initial and Secondary Treatment:

(1) Initially treated patients: patients who have not undergone anti-tuberculosis treatment or have been treated for less than 1 month.
(2) Retreated patients: patients who have been on anti-tuberculosis treatment for more than 1 month (Zhang et al., 2016).
(3) Initial treatment/initial drug resistance: tuberculosis patients who have not received anti-tuberculosis treatment in the past or have been treated for less than 1 month, and the tuberculosis bacteria they are infected with are resistant to at least one anti-tuberculosis drug.
(4) Retreatment case: a patient who had been treated for any form of TB before but has initiated treatment again following relapse or default or failure to cure of the 1st regimen (Getnet et al., 2017).

Patients who had received multiple treatments with anti-tuberculosis drugs were excluded. These drugs primarily consist of the 16 medications mentioned in this study.

### Drug resistance determination

According to the WHO definition of drug-resistant tuberculosis, drug resistance patterns are classified as follows (Song et al., 2019; Shibabaw et al., 2020): Monoresistance refers to tuberculosis bacteria infected by patients that are resistant to only one anti-tuberculosis drug; Multiresistance refers to the resistance of tuberculosis bacteria to more than one anti-tuberculosis drug, excluding simultaneous resistance to Isoniazid and Rifampicin; Multidrug resistance (MDR) refers to resistance to Isoniazid and Rifampicin at the same time; Extensively drug-resistant (XDR) refers to MDR that is also resistant to at least one of fluoroquinolones and second-line anti-tuberculosis injections (kanamycin, capreomycin, amikacin). According to expert consensus and treatment guidelines (Chinese Medic al Association, 2023), this study involves 4 first-line oral anti-tuberculosis drugs and 12 other anti-tuberculosis drugs.

### Statistical methods

A database was established using Epidata 3.1, and statistical analysis was performed using SPSS 22.0. Econometric data was represented using M (QL, QU), and comparisons between groups were made using the rank sum test. Counting data was expressed in terms of rate or composition ratio, and comparisons between groups were made using the χ^2^-test. Univariate analysis of influencing factors was conducted using unconditional logistic regression.

### Quality control

Culture and identification of strains were carried out in strict accordance with the “tuberculosis Diagnostic Laboratory Test Procedures”. The sensitivity test was uniformly conducted using the proportional method. The drug resistance rate was calculated based on the first sputum culture isolation of the patient after admission. Operators received unified training, and a dual entry system was used for data entry.

## Results

In this study, 102 patients were initially treated, while 293 patients underwent re-treatment. Out of these patients, there were 283 males and 112 females. The age distribution does not follow a normal distribution, with the minimum age being 18 years old, the maximum age being 80 years old, and the average age being (47.0 ± 19.0) years old. There were 96 smokers and 299 non-smokers included in the study. No statistically significant differences were found in terms of gender, age, and smoking status between the initial and recurrent patients (*p* > 0.05) (Table 1).

**TABLE 1:** Comparison of general conditions of the two groups.

Items		Initially treated patients (*n* = 102)	Retreated patients (*n* = 293)	Z/t/_χ_^2^	*p*
Sex	Male	76 (74.51%)	207 (70.65%)	0.55	0.456
	Female	26 (25.49%)	86 (29.35%)		
Age (years)		44.0 ± 25.0	47.0 ± 17.5	−1.897	0.058
BMI		22.35 ± 2.70	22.29 ± 2.90	−0.094	0.411
Smoking	Yes	29 (28.43%)	67 (22.87%)	1.273	0.259
	No	73 (71.57%)	226 (77.13%)		

When comparing the rates of Monoresistance, Multiresistance, MDR and XDR between initial and recurrent patients, the results showed that only the difference in MDR rate was statistically significant (χ^2^ = 4.939, *p* = 0.026). The MDR rate of patients in the retreatment group (10.2%) was significantly higher than that of patients in the initial treatment group (2.9%) (Table 2).

**TABLE 2:** Comparison of drug resistance among patients.

Drug resistance types	Initially treated patients (*n* = 102)	Retreated patients (*n* = 293)	_χ_^2^	*p*
Monoresistance	26 (25.5%)	85 (29.0%)	0.464	0.496
Multiresistance	44 (43.1%)	148 (50.5%)	1.647	0.199
MDR	3 (2.9%)	3010.2 (%)	4.939	0.026
XDR	2 (2.0%)	5 (1.7%)	0.028	0.867

Among the first-line oral antituberculosis drugs, the drug resistance rate in the first treatment group was highest for Isoniazid (15.7%), followed by Rifampicin (6.9%) and Ethambutol and Rifabutin (3.9%). In the retreatment group, the highest resistance rates were observed for Isoniazid (24.6%), Rifampicin (17.4%), Rifabutin (11.3%), and Ethambutol (6.5%). The drug resistance rate to Rifampicin and Rifabutin was significantly higher in retreated patients compared to untreated patients (*p* = 0.010, *p* = 0.028). Among second-line oral antituberculosis drugs, the resistance rate to propafenicotinide was higher in retreated patients (63.1%) compared to initially treated patients (46.1%), with a statistically significant difference (*p* = 0.003). There was no statistically significant difference (*p* > 0.05) in the resistance of initially treated patients to other anti-tuberculosis drugs (Table 3).

**TABLE 3:** 16 types of anti-tuberculosis drug resistance in two groups of patients.

Drug sensitivity	Initially treated patient (n = 102)		Retreated patients (n = 293)		Total (%)	χ^2^	*P*
	Drug resistance	Rate (%)	Drug resistance	Rate (%)			
Ciprofloxacin	8	7.8	29	9.9	37 (9.4)	0.376	0.054
Amikacin	1	1.0	3	1.0	4 (1.0)	0.000	1.000
Capreomycin	2	2.0	4	1.4	6 (1.5)	0.000	1.000
Propylthioisoniazid	47	46.1	185	63.1	232 (58.7)	9.087	0.003
Isoniazid Aminosalicylate Tablets	16	15.7	26	8.9	42 (10.6)	3.695	0.055
Moxifloxacin	17	16.7	68	23.2	85 (21.5)	1.917	0.166
Clarithromycin	11	10.8	27	9.2	38 (9.6)	0.242	0.623
Rifabutin	4	3.9	33	11.3	37 (9.4)	4.803	0.028
Ethambutol	4	3.9	19	6.5	23 (5.8)	0.906	0.341
Isoniazid	16	15.7	72	24.6	88 (22.3)	3.451	0.063
Rifampicin	7	6.9	51	17.4	58 (14.7)	6.714	0.010
Streptomycin	19	18.6	74	25.3	93 (23.5)	1.847	0.174
Levofloxacin	9	8.8	26	8.9	35 (8.9)	0	0.988
Ofloxacin	14	13.7	61	20.8	75 (19.0)	2.475	0.116
Linezolid	6	5.9	22	7.5	28 (7.1)	0.303	0.582
Gatifloxacin	7	6.9	16	5.5	23 (5.8)	0.271	0.603

The factors examined in this study to determine their impact on the rate of multidrug resistance were sex, age, nationality, occupation, marital status, presence of diabetes, initial treatment and retreatment history, general health status, BCG vaccine vaccination, smoking, and alcohol consumption. Results from the single factor logistic regression analysis revealed that only the treatment history (i.e., initial treatment and retreatment) was found to be significantly associated with multidrug resistance (*p* = 0.032). The odds ratio (OR) and 95% confidence interval (CI) were calculated to be 3.764 (1.123, 12.612), and the 95% CI did not include the value 1. This suggests that retreatment is a risk factor for the development of multidrug resistance in patients. Furthermore, patients who undergo retreatment are 3.764 times more likely to develop multidrug resistance compared to those who receive initial treatment (Table 4).

**TABLE 4:** Univariate logistic regression analysis of multidrug resistance.

Variables		PR (%)	Waldχ^2^	P	OR	95% CI for OR	
						Lower	Upper
Sex	Male	71.6	0.067	0.795	1.000		
	Female	28.4			1.108	0.510	2.410
Age	<40	31.6	4.575	0.102	1.000		
	40–49	30.4			0.487	0.200	1.183
	≥50	38.0			0.435	0.185	1.022
BMI			4.082	0.167	0.822	0.156	4.335
Nationality	Han	93.7	3.026	0.388	1.000		
	Manchu	3.8			1.809	0.389	8.406
	Korean	1.5			2.352	0.266	20.809
	Other	0.8			5.879	0.517	66.796
Occupation	Farmer	58.0	4.736	0.192	1.000		
	Worker	2.5			1.479	0.176	12.416
	Unemployed	29.9			1.939	0.923	4.074
	Other	9.6			0.360	0.046	2.795
Marital status	Unmarried	9.1	5.339	0.149	1.000		
	Married	70.9			0.784	0.257	2.398
	Widow	18.0			0.232	0.040	1.332
	Divorced	2.0			2.667	0.396	17.977
Diabetes	No	89.4	0.766	0.381	1.000		
	Yes	10.6			0.519	0.120	2.252
TB treatment	Initial	25.8	4.617	0.032	1.000		
	Retreated	74.2			3.764	1.123	12.612
Health condition	Good	11.6	5.000	0.082	1.000		
	Common	75.7			0.588	0.209	1.652
	Poor	12.7			1.562	0.472	5.172
BCG vaccination	No	84.3	2.291	0.130	1.000		
	Yes	15.7			3.079	0.718	13.214
Smoking	No	75.7	0	0.993	1.000		
	Yes	24.3			0.996	0.434	2.289
Drinking	No	76.7	0.981	0.322	1.000		
	Yes	23.3			1.485	0.679	3.246

## Discussions

The 2021 WHO Global Tuberculosis Report provides a grim assessment of the global tuberculosis incidence. The annual decline in tuberculosis incidence rates has stalled or even reversed, and the estimated death toll from tuberculosis has increased (Chakaya et al., 2022). Although there are differences in prevention and treatment models both domestically and internationally, drug-resistant tuberculosis clearly imposes a heavy economic burden on patients’ families and increases the risk factors for social stability (Devoid et al., 2022; Jarde et al., 2022; Trauer, 2023). The plan to eradicate tuberculosis still requires significant efforts (Aia et al., 2022). Through an analysis of drug resistance in tuberculosis patients admitted to Jilin Provincial Tuberculosis Hospital in 2023, several findings were made. Comparing the rates of single drug resistance, multi-drug resistance, and broad drug resistance between patients undergoing initial and retreatment, it was found that the multi-drug resistance rate among retreatment patients (10.2%) was significantly higher than that among patients receiving initial treatment (2.9%). These findings align with results reported in other parts of the world. For instance, a meta-analysis of 18,908 tuberculosis patients across 24 studies showed a slight variation in drug resistance rates between initial treatment (2.64%) and retreatment (11.54%) (Reta et al., 2022). In a study of 207 tuberculosis patients in Osun State (Oyedeji et al., 2020), Nigeria, the prevalence rates of multi-drug resistant tuberculosis in previously treated and new cases were 7.0% and 3.5%, respectively. Similarly, the first national survey of tuberculosis drug resistance in Lao People’s Democratic Republic revealed a retreatment multi-drug resistance rate of 2.3%, whereas the rate for initial treatment was 0.5% (Iem et al., 2019). Another study conducted in Basra, Iraq, involving 2,542 new and old patients found that the drug resistance rate among retreatment patients was significantly higher than that among initially diagnosed patients (20.3% vs. 2.4%) (Mohammed et al., 2022). These findings suggest that retreatment typically results in higher drug resistance rates in underdeveloped regions, which is consistent with the conclusions of this study. Comparing these results with those from domestic studies, the retreatment multi-drug resistance rate among 236 tuberculosis cases in Huairou District of Beijing was found to be higher at 34.5%, as opposed to 6.8% for initial treatment. This discrepancy may be attributed to differences in population density and drug use, as the capital city has a much higher population density than Jilin Province (Zhang et al., 2021). Finally, comparing the results to Dalian City in Liaoning Province, another city in northeastern China, this study found lower rates of initial multidrug resistance (2.9%) and retreatment multidrug resistance (10.2%) compared to Dalian City’s rates of 5.8% and 17.7%, respectively. This indicates that Jilin Province has a lower drug resistance rate and demonstrates effective control measures (Ganapathi et al., 2017; Wang et al., 2019). Overall, the drug resistance rates of both initially diagnosed and retreatment cases have decreased in recent years, but the decrease is more significant in initially diagnosed cases, while the drug resistance rate among retreatment cases continues to rise (Duan et al., 2016). Insufficient public health resources, lack of public attention, and noncompliance with treatment regulations have contributed to the difficulty in treating tuberculosis, and retreatment cases are more likely to develop into multidrug-resistant cases.

Four first-line oral antituberculosis drugs have been identified, with the following drug resistance rates: Isoniazid (22.3%), Rifampicin (14.7%), Rifabutin (9.4%), and Ethambutol (5.8%). The success of TB prevention efforts in Jilin Province in recent years means that the survey results can inform the selection of clinical drugs in the area. It also provides a foundation for treating patients in the region and controlling drug-resistant tuberculosis. The drug resistance rates for Rifampicin and Rifabutin were significantly higher among retreated patients compared to untreated patients (*p* = 0.010, *p* = 0.028, respectively). This suggests that Rifampicin and Rifabutin may be more suitable for treating newly diagnosed pulmonary tuberculosis patients. The resistance rate of retreated patients to propafenamide, a second-line oral antituberculosis drug, was significantly higher than that of newly diagnosed patients (63.1% vs. 46.1%). This indicates that it may be advisable to avoid using highly resistant drugs in future clinical treatments for retreated patients. This study confirms that treatment history, including initial and recurrent treatment, is the only factor associated with multidrug resistance when using univariate logistic regression analysis. Recurrent patients are 3.764 times more likely to have multidrug resistance compared to initial treatment patients, regardless of gender, age, or smoking status. Other studies have shown that factors such as age<30 years, unemployment rate, economic status, residence, lifestyle, and previous treatment of tuberculosis are also related to the occurrence of multidrug-resistant tuberculosis (MDR-TB) (Ali et al., 2019; Chakaya et al., 2021; Lecai et al., 2021). The development of initially treated drug-resistant tuberculosis into retreatment drug-resistant tuberculosis can be influenced by various factors, including long treatment cycles, improper use of antituberculosis drugs, drug side effects, poor patient compliance, economic difficulties, and other reasons. Additionally, the production of drug-resistant strains, low patient immunity, and comorbidities such as diabetes or other pulmonary infections are also factors that cannot be ignored (Shivekar et al., 2020; Antonio-Arques et al., 2021; Antimicrobial Resistance Collaborators, 2022; Williams et al., 2022). However, this study did not find an association between BMI and binding resistance, while previous studies have shown that higher BMI was negatively associated with being a relapse/defaulter/treatment-failure cases (Goswami et al., 2014; Sharma et al., 2019). Improved nutritional status, which can positively influence immunity and treatment outcome, could be cited as a possible explanation; but their findings could also have been an artifact of reverse causation, as implicated by deterioration of general health among relapse/defaulter/treatment-failure cases. This study failed to establish a correlation between BMI and tuberculosis resistance, potentially due to the absence of long-term BMI changes in patients. Hence, additional research is required to ascertain the relationship between BMI and tuberculosis resistance.

To sum up, timely treatment and standardized management of newly diagnosed tuberculosis patients are crucial in preventing the occurrence of drug-resistant tuberculosis. Medical institutions should improve relevant regulations and systems (Pontali et al., 2019; Williams et al., 2022), establish a new tuberculosis monitoring system (Jiang et al., 2021), and collaborate with relevant departments to develop social and economic models and programs. Furthermore, collective efforts are needed to increase awareness of tuberculosis (Katiyar and Katiyar, 2019; Long et al., 2020). A program tailored to our socio-economic conditions is necessary to enhance tuberculosis management by private sectors, promote unity in treatment, provide better treatment outcomes, and help prevent the spread of the disease within the community while inhibiting the development of drug resistance.

## Conclusion

The prevalence of multidrug-resistant is higher in retreated patients compared to initially treated patients in the study population. Multidrug resistance is only associated with the treatment history (initial retreatment) and not with other factors.

## Data Availability

The original contributions presented in the study are included in the article/Supplementary Material, further inquiries can be directed to the corresponding author.
